# Supplementary material for: Structure of the MUC5AC VWD3 assembly responsible for the formation of net-like mucin polymers
Source: EMBO Rep. 2025 Feb 27;26(6):1457–71. doi: 10.1038/s44319-025-00395-8 (PMC11933400; doi:10.1038/s44319-025-00395-8)
Supplement: Supplementary file 1 — Appendix [file 44319_2025_395_MOESM1_ESM.pdf]

APPENDIX to Structure of the MUC5AC VWD3 assembly responsible for the formation of net-like mucin polymers

SUPPLEMENTARY FIGURES AND TABLE

Page 2, Appendix Figure S1. Cryo-EM structure of MUC5AC-D´3D3CysD1.
Page 3, Appendix Figure S2. Cryo-EM map of MUC5AC-D´3D3CysD1 open conformation.
Page 4, Appendix Figure S3. Cryo-EM structure of MUC5AC-D´3D3CysD1 R996Q.
Page 5, Appendix Figure S4. Cryo-EM structure of MUC5AC-D3 R1201W.
Page 6, Appendix Figure S5. Cryo-EM structure of MUC5AC-D3 R996Q R1201W.
Page 7, Appendix Figure S6. Cryo-EM structure of MUC5AC-6xHis-D3 R996Q.
Page 8, Appendix Figure S7. MUC5AC cryo-EM maps of colored by local resolution.
Page 9, Appendix Table S1. MUC5AC cryo-electron microscopy parameters.

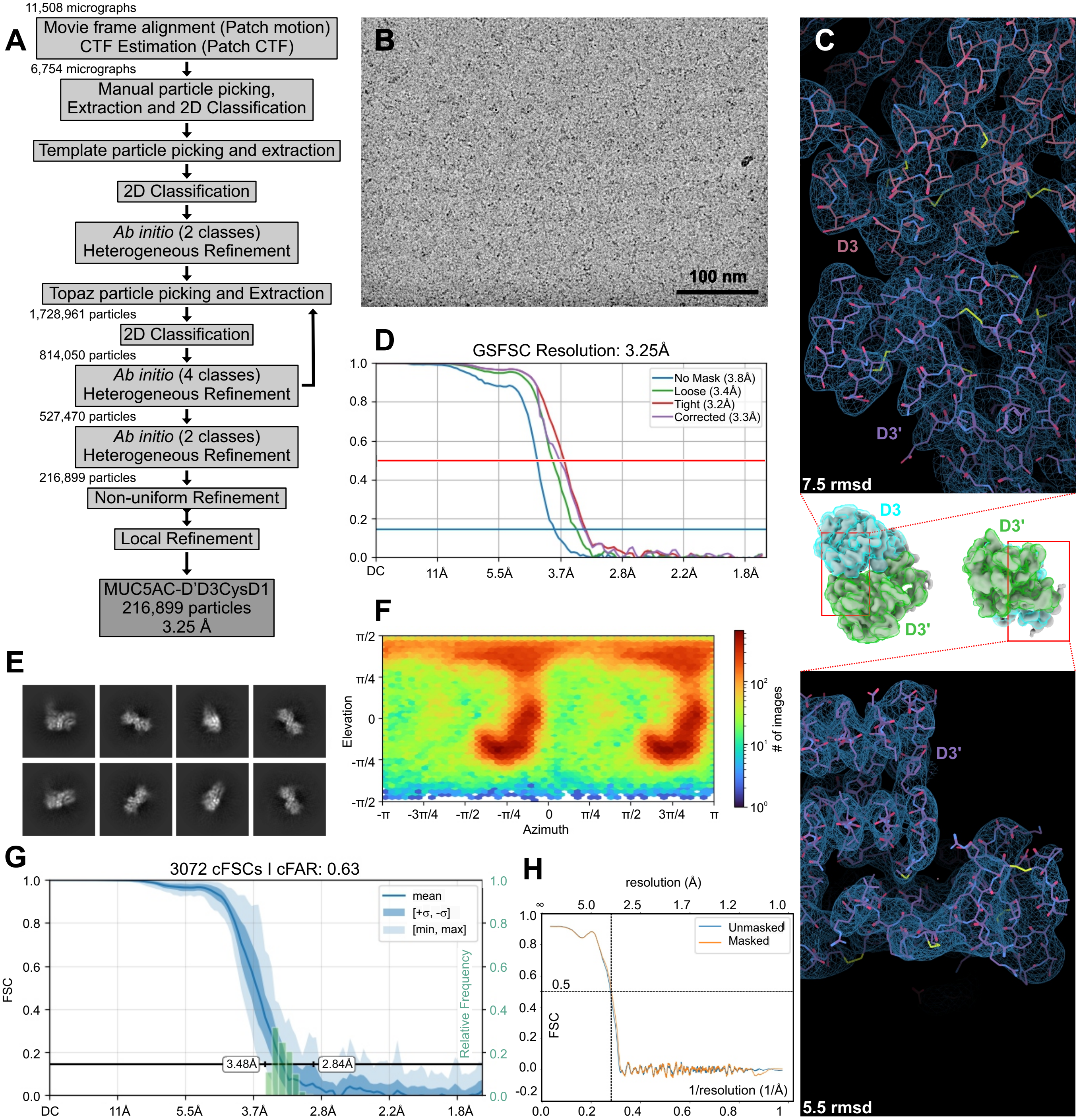

Appendix Figure S1. Cryo-EM structure of MUC5AC-D´3D3CysD1.

(A) Flowchart summary of Cryo-EM processing steps. The number of micrographs used and the number of particles remaining after each sorting step for the last iteration are specified.
(B) Representative micrograph. Scale bar is shown.
(C) Map-model fitting overview.
(D) Fourier Shell Correlations (FSC) for the final density map.
(E) Representative 2D classes.
(F) Per-particle distribution over azimuth and elevation angles for the final density map.
(G) Conical FSC Area Ratio (cFAR) evaluated with respect to 3072 viewing directions.
(H) FSC (Model-map).

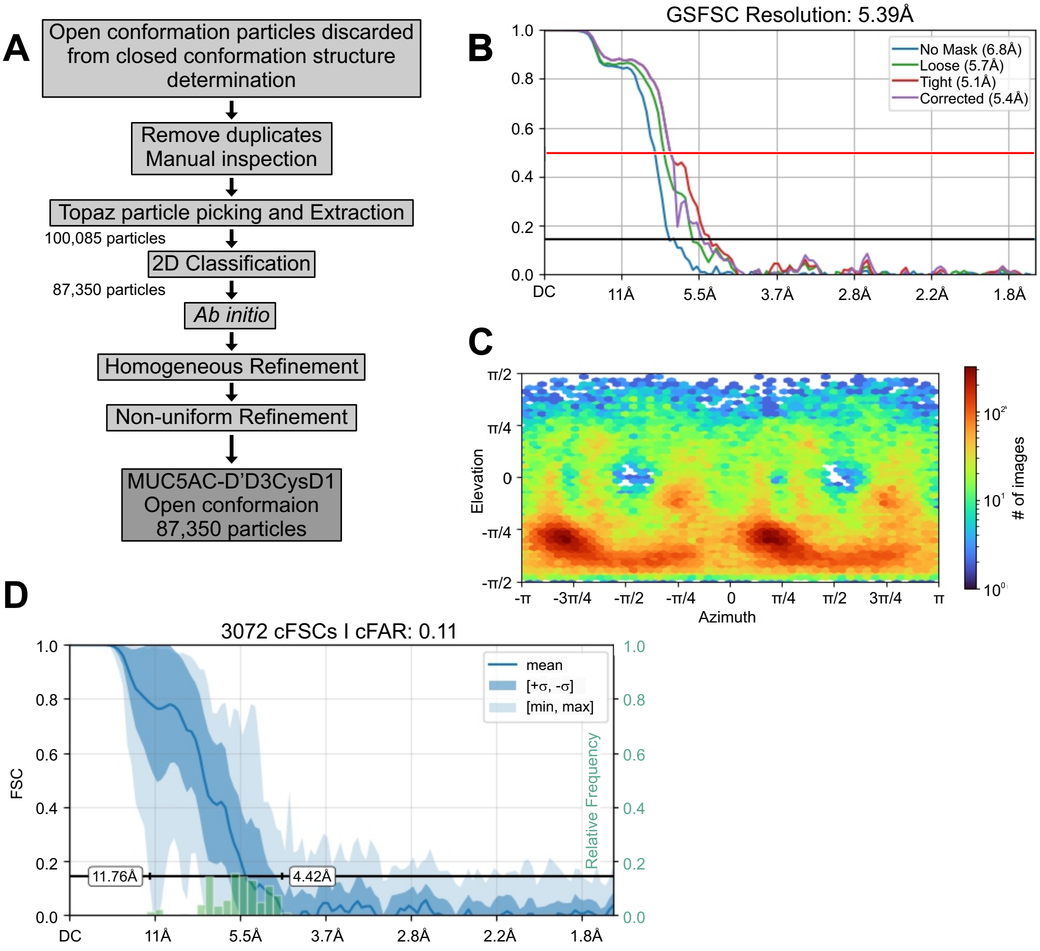

Appendix Figure S2. Cryo-EM map of MUC5AC-D´3D3CysD1 open conformation.
(A) Flowchart summary of Cryo-EM processing steps.
(B) Fourier Shell Correlations (FSC) for the final density map.
(C) Per-particle distribution over azimuth and elevation angles for the final density map.
(D) Conical FSC Area Ratio (cFAR) evaluated with respect to 3072 viewing directions.

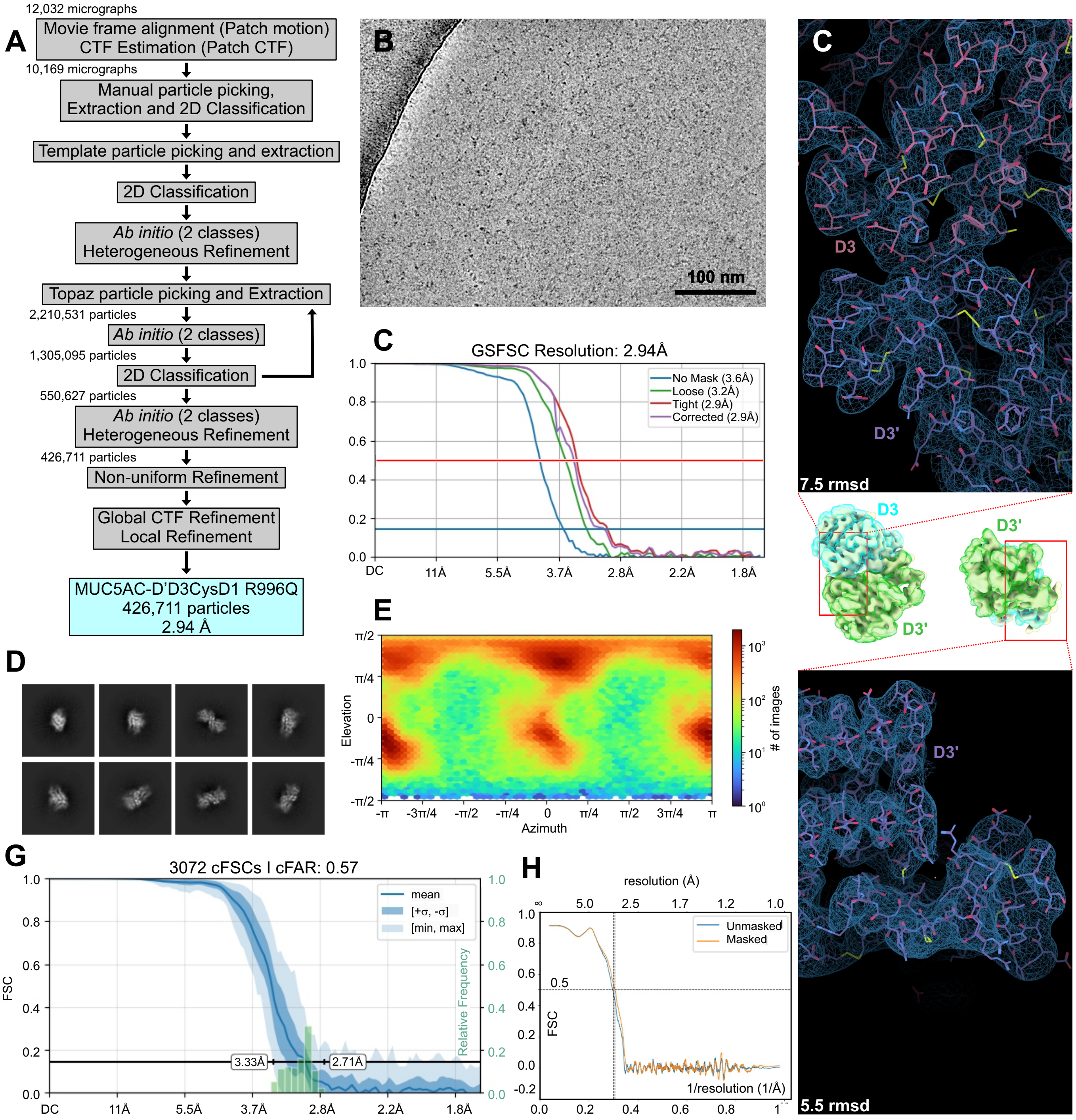

Appendix Figure S3. Cryo-EM structure of MUC5AC-D´3D3CysD1 R996Q.
(A) Flowchart summary of Cryo-EM processing steps. The number of micrographs used and the number of particles remaining after each sorting step for the last iteration are specified.
(B) Representative micrograph. Scale bar is shown.
(C) Map-model fitting overview.
(D) Fourier Shell Correlations (FSC) for the final density map.
(E) Representative 2D classes.
(F) Per-particle distribution over azimuth and elevation angles for the final density map.
(G) Conical FSC Area Ratio (cFAR) evaluated with respect to 3072 viewing directions.
(H) FSC (Model-map).

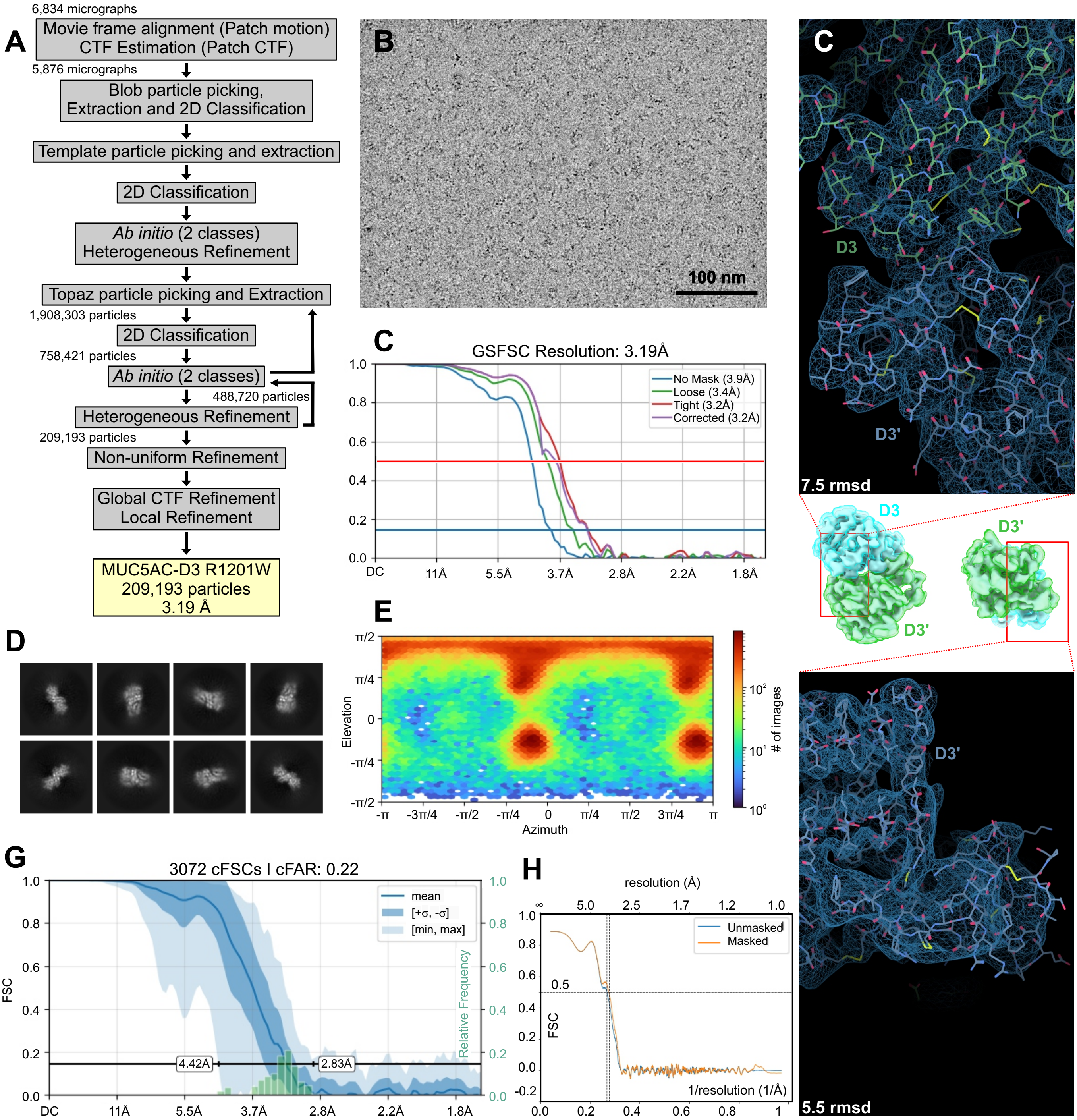

Appendix Figure S4. Cryo-EM structure of MUC5AC-D3 R1201W.
(A) Flowchart summary of Cryo-EM processing steps. The number of micrographs used and the number of particles remaining after each sorting step for the last iteration are specified.
(B) Representative micrograph. Scale bar is shown.
(C) Map-model fitting overview.
(D) Fourier Shell Correlations (FSC) for the final density map.
(E) Representative 2D classes.
(F) Per-particle distribution over azimuth and elevation angles for the final density map.
(G) Conical FSC Area Ratio (cFAR) evaluated with respect to 3072 viewing directions.
(H) FSC (Model-map).

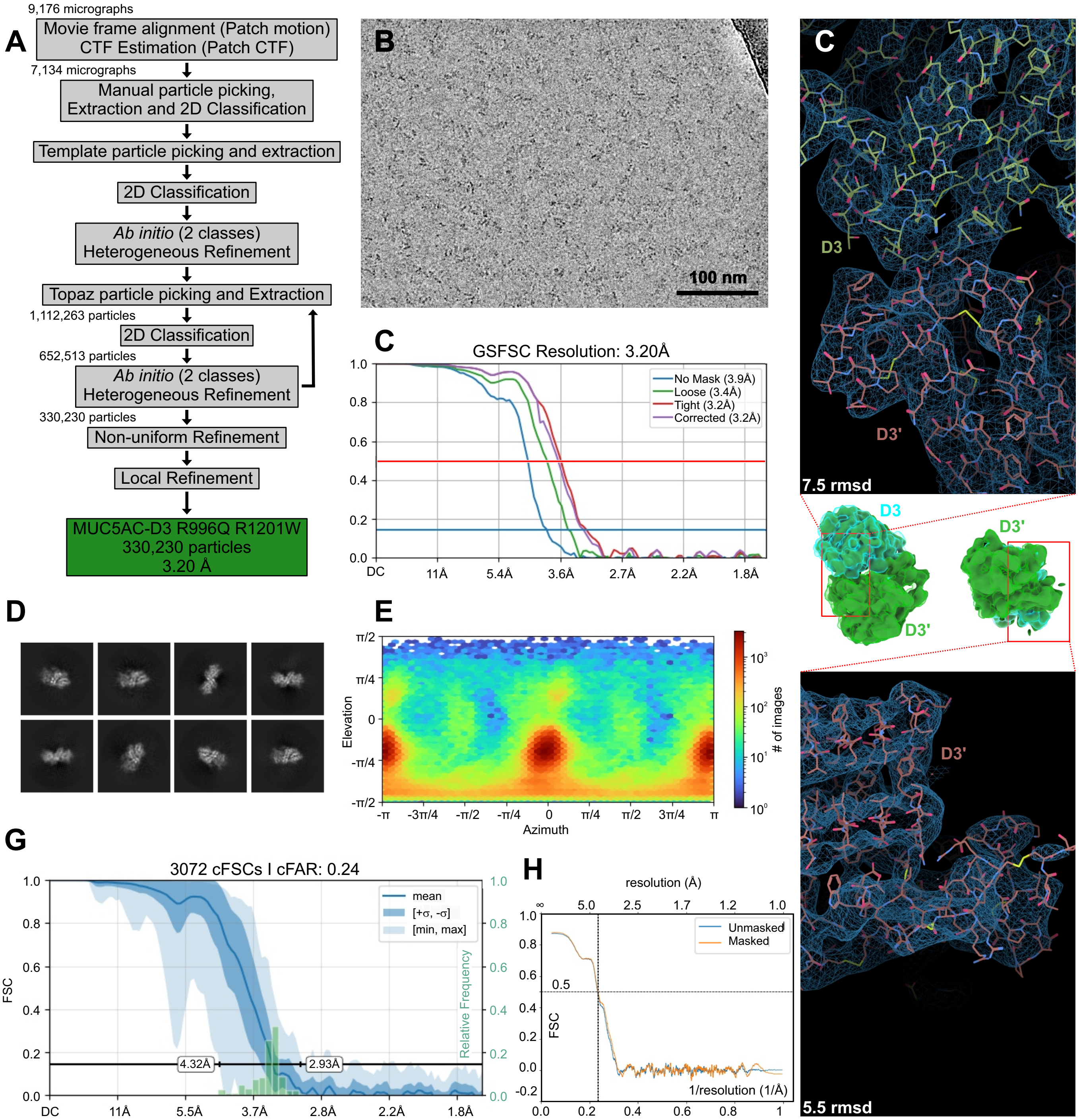

Appendix Figure S5. Cryo-EM structure of MUC5AC-D3 R996Q R1201W.
(A) Flowchart summary of Cryo-EM processing steps. The number of micrographs used and the number of particles remaining after each sorting step for the last iteration are specified.
(B) Representative micrograph. Scale bar is shown.
(C) Map-model fitting overview.
(D) Fourier Shell Correlations (FSC) for the final density map.
(E) Representative 2D classes.
(F) Per-particle distribution over azimuth and elevation angles for the final density map.
(G) Conical FSC Area Ratio (cFAR) evaluated with respect to 3072 viewing directions.
(H) FSC (Model-map).

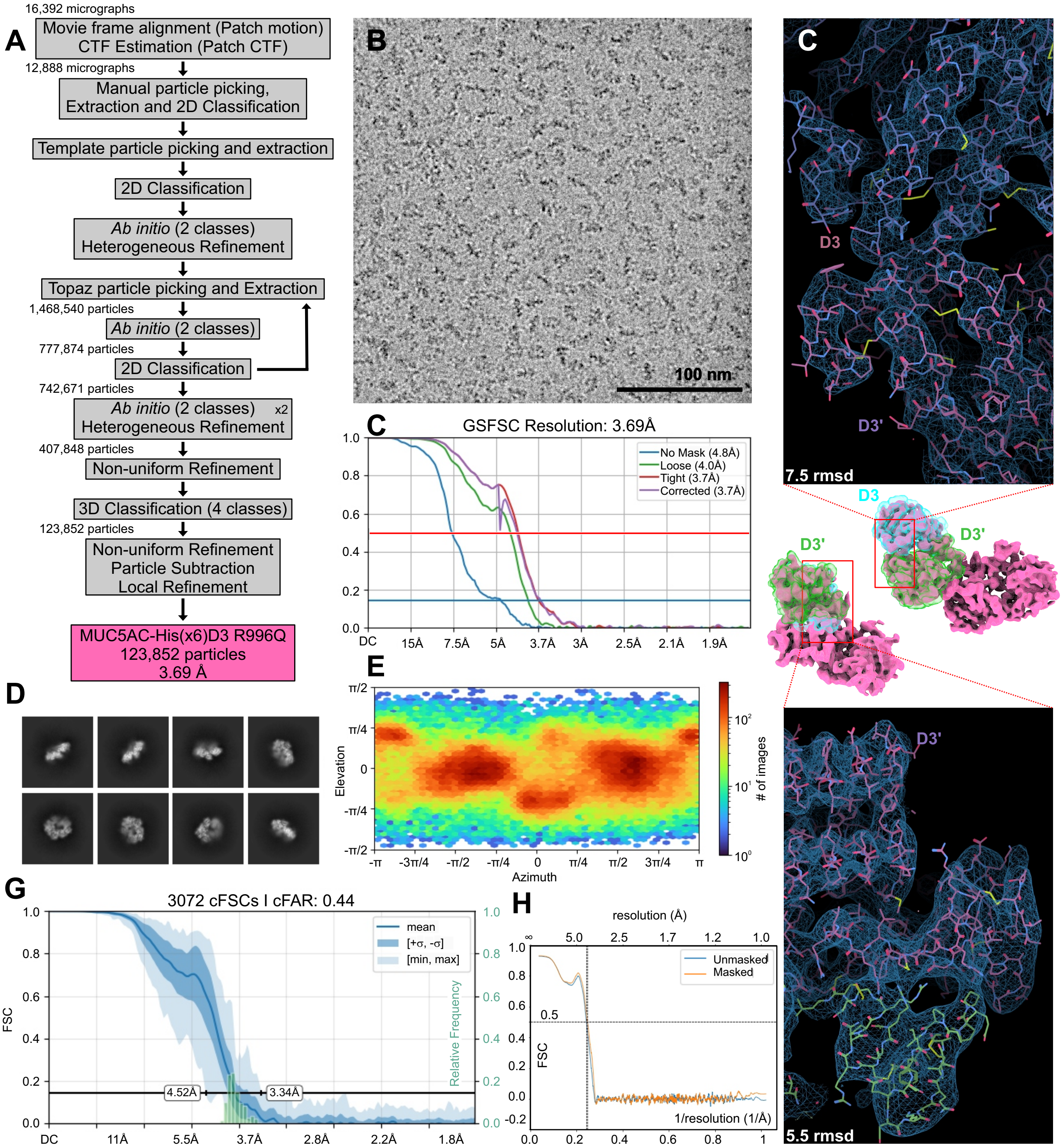

Appendix Figure S6. Cryo-EM structure of MUC5AC-6xHis-D3 R996Q.

(A) Flowchart summary of Cryo-EM processing steps. The number of micrographs used and the number of particles remaining after each sorting step for the last iteration are specified.

(B) Representative micrograph. Scale bar is shown.

(C) Map-model fitting overview.

(D) Fourier Shell Correlations (FSC) for the final density map.

(E) Representative 2D classes.

(F) Per-particle distribution over azimuth and elevation angles for the final density map.

(G) Conical FSC Area Ratio (cFAR) evaluated with respect to 3072 viewing directions.

(H) FSC (Model-map).

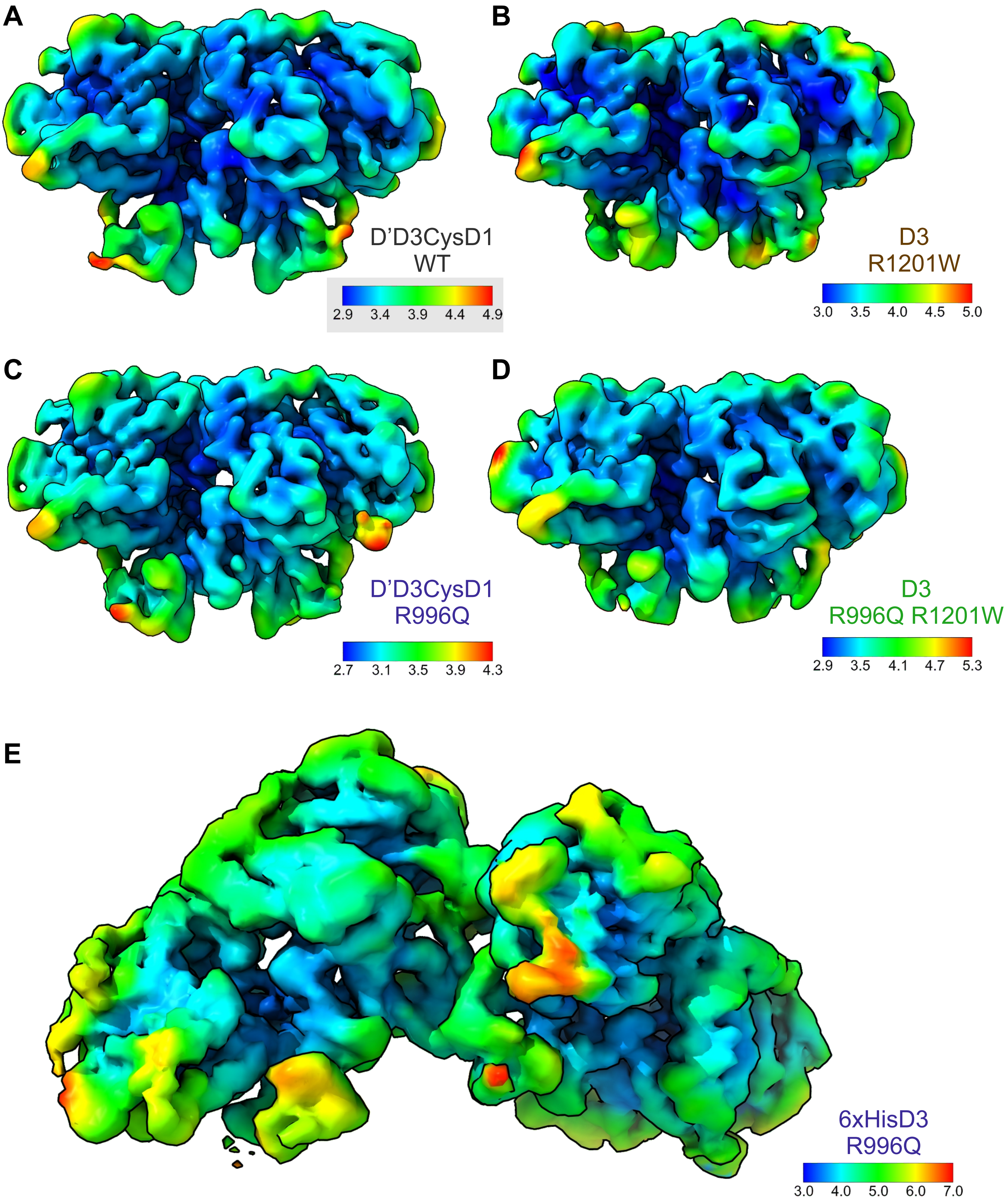

Appendix Figure S7. MUC5AC cryo-EM maps of colored by local resolution.
The color keys show local resolution in Angstrom (Å). The keys were automatically generated showing the full range resolution calculated at FSC threshold 0.5.
(A) MUC5AC D’D3CysD1 dimer.
(B) MUC5AC D3 R1201W dimer.
(C) MUC5AC D’D3CysD1 R996Q dimer.
(D) MUC5AC D3 R996Q R1201W dimer.
(E) MUC%AC 6xHisD3 R996Q tetramer.

Appendix Table S1. MUC5AC cryo-electron microscopy parameters.

**Structure**	**D'D3CysD1**	**D'D3CysD1 R996Q**	**D3 R1201W**	**D3 R996Q R1201W**	**His(x6)D3 R996Q**
**Data Accession**					
PDB	8QTV	8QTB	8R1U	8R1Z	8QSP
EMDB	EMD-18654	EMD-18648	EMD-18828	EMD-18829	EMD-18638
**Data Collection**					
Microscope	TFS KRIOS	TFS KRIOS	TFS KRIOS	TFS KRIOS	TFS KRIOS
Voltage (kV)	300	300	300	300	300
Detector	GATAN K3	GATAN K3	GATAN K3	GATAN K3	GATAN K2
Pixel Size (Å)	0.86	0.86	0.86	0.86	0.83
Electron exposure ($e^- /Å^2$)	50	51.6	50	50.3	46.2
Defocus range (µm)	-0.5 to -2.5	-0.5 to -3.0	-0.5 to -3.0	-0.5 to -2.5	-0.5 to -3.5
Micrographs	11,508	12,032	6,834	9,176	16,392
**Reconstruction**					
Software	CryoSPARC (v3.2)	CryoSPARC (v3.2)	CryoSPARC (v3.2)	CryoSPARC (v3.2)	CryoSPARC (v3.2)
Micrographs used	6,754	10,169	5,876	7,134	12,888
Particles used in refinement	216,899	426,711	209,193	330,230	123,852
Symmetry imposed	C2	C2	C2	C2	C1
Overall resol. (Å)					
FSC=0.143 (masked)	3.25	2.94	3.19	3.20	3.69
Map sharpening B-factor ($Å^2$)	-168.4	-141.6	-152.4	-167.5	-122.1
Local resol. rang.(Å)	2.869-5.503	2.685-5.164	2.889-6.583	2.896-47.60	3.275-10.43
**Model Refinement**					
Software	Phenix (v1.20.1-4487)	Phenix (v1.20.1-4487)	Phenix (v1.20.1-4487)	Phenix (v1.20.1-4487)	Phenix (v1.20.1-4487)
Non-hydrogen atoms	5,054	5,050	5,032	5,028	10,174
Protein residues	662	662	658	658	1,333
Ligands	2	2	2	2	4
Av. B factors ($Å^2$)					
Protein	184.06	171.86	183.73	206.34	190.77
Ligands	253.47	215.50	216.35	55.94	213.07
R.M.S. deviations					
Bond length (Å)	0.002	0.002	0.002	0.003	0.002
Bond angle (°)	0.429	0.405	0.455	0.484	0.487
Ramachandran statistics (%)					
Outliers	0.00	0.00	0.00	0.00	0.00
Allowed	5.17	2.74	4.89	5.2	4.15
Favored	94.83	97.26	95.11	94.9	95.85
MolProbity score	1.56	1.27	1.61	1.93	1.83
Model vs. Map FSC					
FSC=0.5 (masked,Å)	3.5	3.3	3.6	4.2	4.1
