# Supplementary figures and images for: Structure of the MUC5AC VWD3 assembly responsible for the formation of net-like mucin polymers
Source: EMBO Rep. 2025 Feb 27;26(6):1457–71. doi: 10.1038/s44319-025-00395-8 (PMC11933400; doi:10.1038/s44319-025-00395-8)

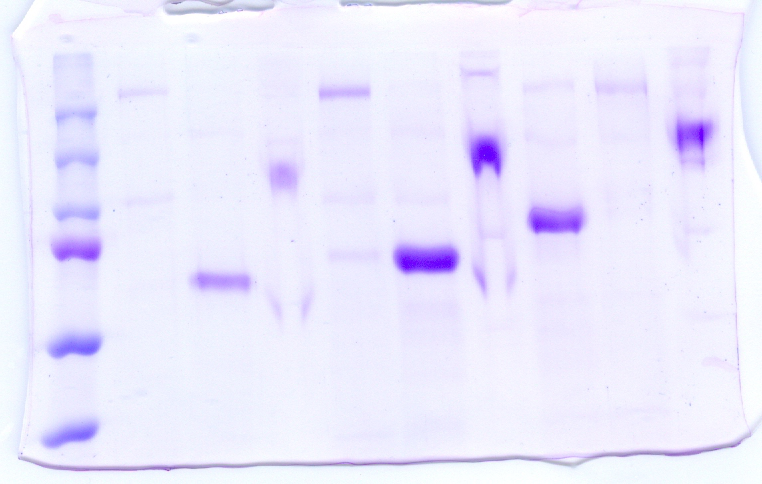

Supplement: Supplementary file 3 — Source data Fig. 1 [file 44319_2025_395_MOESM3_ESM.zip › Figure 1C/Fig 1C.tif]

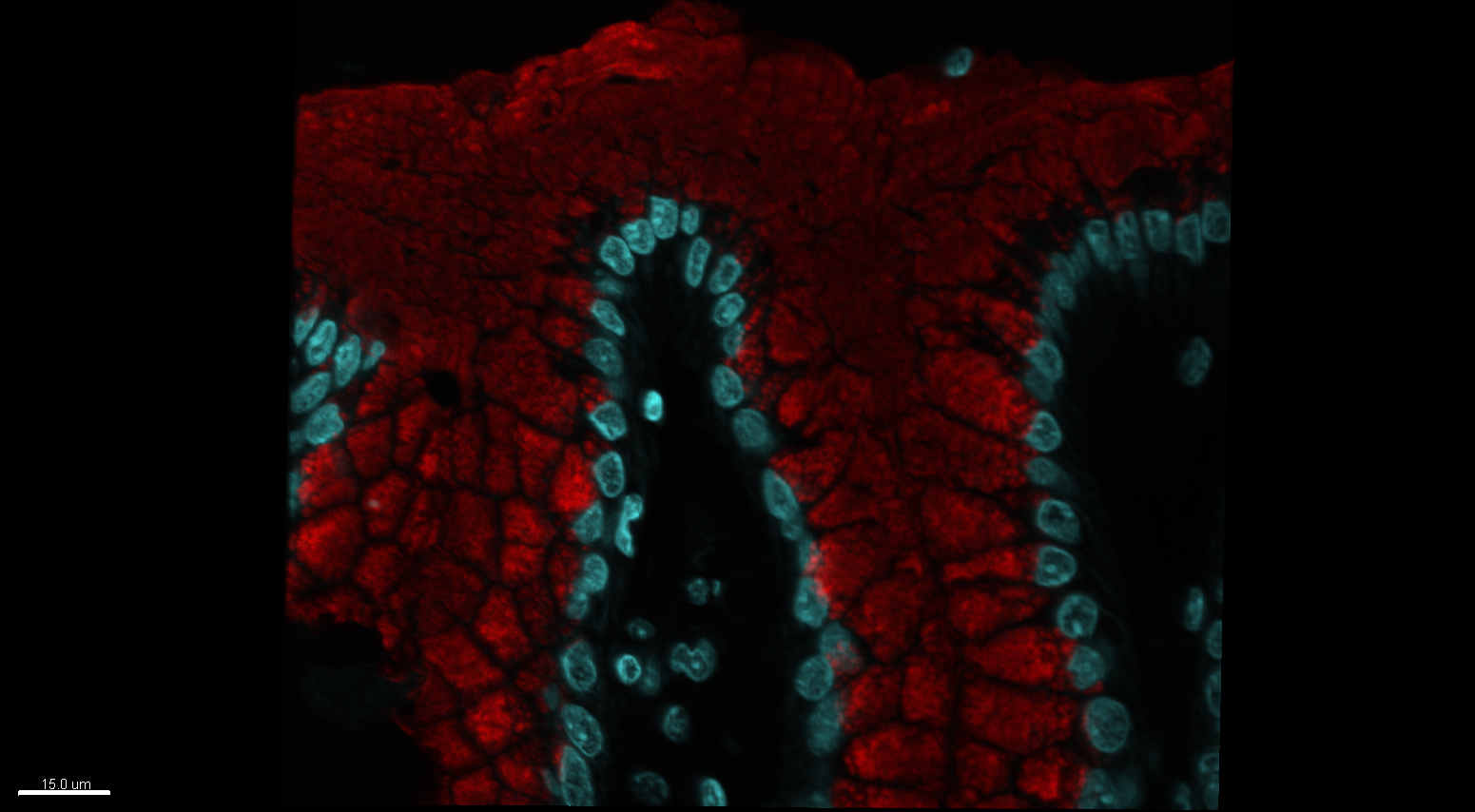

Supplement: Supplementary file 4 — Source data Fig. 5 [file 44319_2025_395_MOESM4_ESM.zip › Figure 5/Fig 5C.tif]
